# Improving symptom burden in adults with persistent post-concussive symptoms: a randomized aerobic exercise trial protocol

**DOI:** 10.1186/s12883-020-1622-x

**Published:** 2020-02-05

**Authors:** Leah J. Mercier, Tak S. Fung, Ashley D. Harris, Sean P. Dukelow, Chantel T. Debert

**Affiliations:** 1 Department of Clinical Neurosciences, Division of Physical Medicine and Rehabilitation, University of Calgary, Calgary, AB Canada; 2 Information Technologies, University of Calgary, Calgary, AB Canada; 3 Department of Radiology, University of Calgary, Calgary, AB Canada

**Keywords:** Concussion, Mild traumatic brain injury, Persistent post-concussive symptoms, Aerobic exercise, Randomized controlled trial, Exercise intolerance, Post-concussion syndrome, Quality of life

## Abstract

**Background:**

Persistent post-concussive symptoms (PPCS) affect up to 30% of individuals following mild traumatic brain injury. PPCS frequently includes exercise intolerance. Sub-symptom threshold aerobic exercise has been proposed as a treatment option for symptom burden and exercise intolerance in this population. The primary aim of this study is to evaluate whether a progressive, sub-symptom threshold aerobic exercise program can alleviate symptom burden in adults with PPCS.

**Methods:**

Fifty-six adults (18–65) with PPCS (>3mos-5 yrs) will be randomized into two groups: an immediate start 12-week aerobic exercise protocol (AEP) or delayed start 6-week placebo-like stretching protocol (SP), followed by AEP. Aerobic or stretching activities will be completed 5x/week for 30 mins during the intervention. Online daily activity logs will be submitted. Exercise prescriptions for the AEP will be 70–80% of heart rate at the point of symptom exacerbation achieved on a treadmill test with heart rate monitoring. Exercise prescription will be updated every 3-weeks with a repeat treadmill test. The Rivermead Post-concussion Symptom Questionnaire will be the primary outcome measure at 6 and 12-weeks of intervention. Secondary outcomes include assessments of specific symptoms (headache, quality of life, mood, anxiety, fatigue, dizziness, sleep parameters, daytime sleepiness) in addition to blood biomarkers and magnetic resonance imaging and spectroscopy data for quantification of brain metabolites including γ-aminobutyric acid (GABA), glutathione, glutamate and N-acetyl aspartate (NAA) all measured at 6 and 12-weeks of intervention.

**Discussion:**

This trial will evaluate the use of aerobic exercise as an intervention for adults with PPCS, thus expanding our knowledge of this treatment option previously studied predominantly for adolescent sport-related concussion.

**Trial registration:**

ClinicalTrials.gov - NCT03895450 (registered 2019-Feb-11).

## Background

Concussion, a term often used interchangeably with mild traumatic brain injury (mTBI), results from a biomechanical force to the head inducing a sequelae of homeostatic disruptions and biochemical alterations [1]. Of all treated brain injuries, approximately 80% are classified as mild [2]. While the majority of individuals recover from mTBI in the 10–14 day acute period [3], a minority will go on to experience persistent post-concussive symptoms (PPCS) greater than 3 months. PPCS are heterogenous, but frequently include headache, dizziness, cognitive difficulties, fatigue, low mood, anxiety, emotional lability, exercise intolerance and sleep disturbances. Headache and cognitive difficulties (poor memory, difficulty concentrating) are among the most commonly reported symptoms months to years post-mTBI [4–8], in addition to fatigue [9]. At three months post-mTBI 22–40% of individuals meet ICD-10 criteria for persistent post-concussive symptoms (PPCS) [10, 11], also termed post-concussion syndrome, and up to 33% of individuals are functionally impaired based on Glasgow Coma Score-extended criteria [12].

Consensus on return to activity following mTBI has shifted considerably in recent years. Earlier editions of concussion consensus guidelines suggested rest following mTBI until complete symptom resolution [13]. The risk of a subsequent mTBI during an acute period of physiological vulnerability, which is augmented by slower thinking and reflexes, contributed to this recommendation. Following these guidelines, the notion of “rest is best” was widely adopted and reinforced [13–16]. However, recent studies have found that after a brief period (24–48 h) of rest following mTBI, exercise below symptom threshold may improve recovery over rest [17]. A large cohort study of children and adolescents (age 5 to 17.99) recruited from the emergency department found those who participated in some type of physical activity (light aerobic exercise, sport-specific exercise, noncontact drills or full-contact practice/competition) within 7 days of injury had a lower risk of PPCS at 28 days compared to those not having participated in any physical activity [18]. With new understanding that previous rest recommendations are not always beneficial to recovery, there is a great need for trials evaluating sub-symptom threshold aerobic exercise as a treatment for individuals with PPCS.

Sub-symptom threshold aerobic exercise is aerobic activity below the heart rate (HR) at which an individual’s mTBI-related symptoms are worsened. Exercise intolerance due to autonomic dysfunction is associated with elevated cerebral blood flow and arterial carbon dioxide contributing to onset of symptom exacerbation during exercise [19]. In non-concussed individuals, aerobic exercise has been shown to improve headache frequency [20], fatigue [21], mental health [22], cognition [23] and sleep [24], all of which are characteristic of PPCS. With potential to improve many mTBI-related symptoms, aerobic exercise is a promising treatment option for PPCS, yet research has largely focussed on the pediatric sport-related concussion (SRC) population in the acute/sub-acute phase following injury [25]. Leddy and colleagues investigated a progressive sub-symptom threshold intervention acutely following SRC in adolescents (age 13 to 18). Those randomized to the aerobic exercise intervention were asymptomatic in a median of 13 days, whereas those in the stretching group recovered in a median of 17 days [26]. In another adolescent sample with 4–16 weeks of persistent symptoms Kurowski et al. compared a 9-week sub-symptom aerobic training protocol to a full-body stretching protocol [27]. A greater rate of improvement based Post Concussion Symptom Inventory scores, a measure of symptom burden, was observed in the aerobic exercise group compared to the stretching group. While aerobic exercise trials in adolescents have shown promise, a similar intervention in an adult PPCS cohort has not previously been investigated. Our study participants have heterogeneous injury etiology that is not necessarily sport-related. This study cohort can also be differentiated from previous literature due to the chronic nature of symptoms (> 3 months to years), often producing functional impairment and inability to continue with employment. There is great need for personalized, low cost, non-pharmacological interventions for this adult population with potential to treat multiple post-concussive symptoms, such as aerobic exercise.

This study will investigate the utility of sub-symptom threshold aerobic exercise for the treatment of PPCS using a randomized, placebo controlled, delayed-start design. Our specific objectives are the following:

1. Evaluate whether an aerobic exercise protocol (AEP) produces a clinically meaningful improvement in post-concussive symptom burden compared to a placebo-like stretching protocol (SP).
2. Evaluate change in quality of life and specific post-concussive symptoms, including headache, mood, anxiety, fatigue, dizziness, sleep parameters and daytime sleepiness, following AEP and SP.
3. Evaluate change in blood and imaging biomarkers of exercise in mTBI recovery following AEP and SP to better understand the mechanisms of recovery.

This trial is designed to assess measures of symptom severity and quality of life in addition to investigating the underlying mechanism of recovery following intervention. These measures may help us identify which individuals will best respond to a tailored exercise program, such as that described in this protocol.

## Methods/design

### Study design

**Fig. 1:**
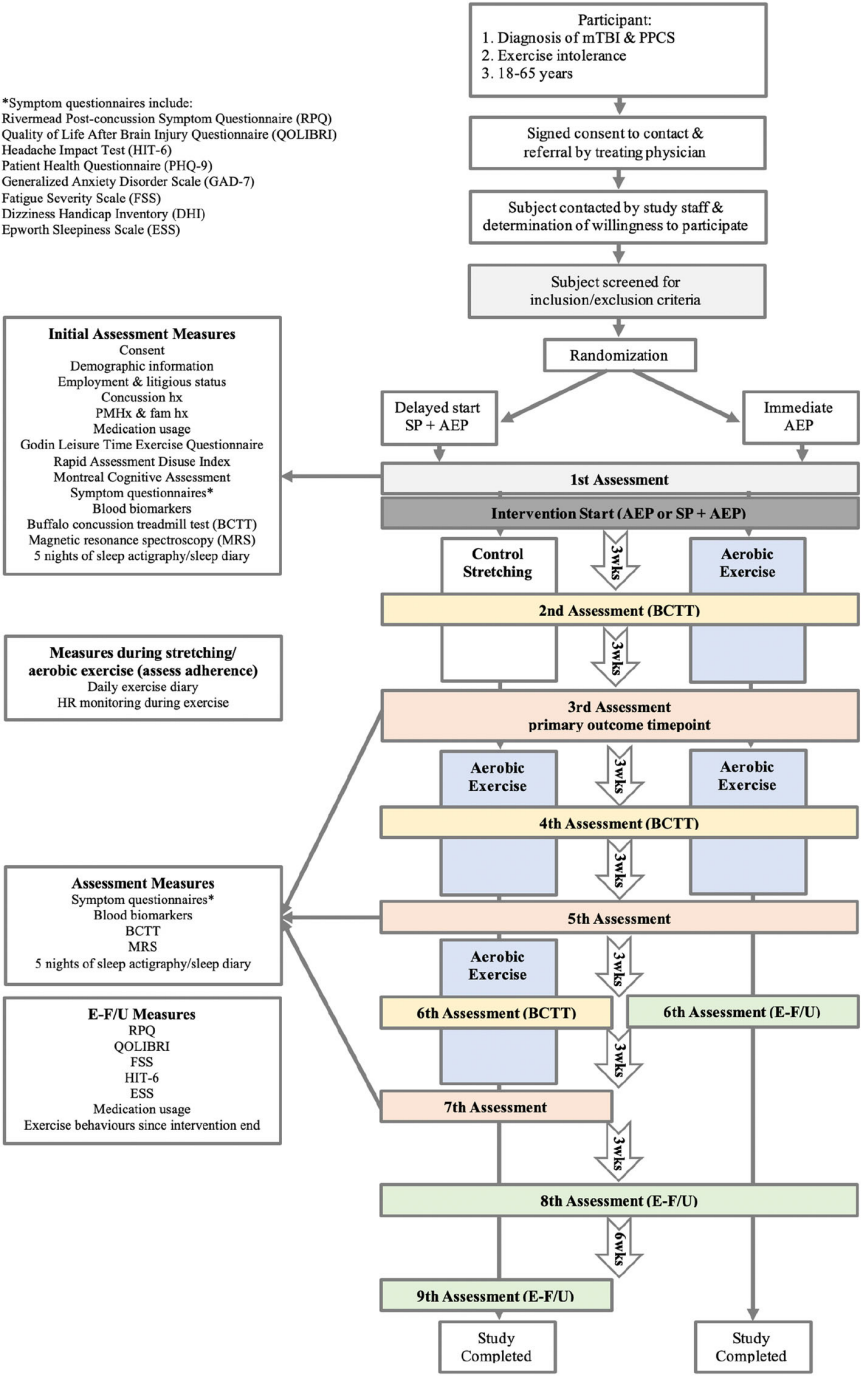
Study Design Protocol

This is a randomized control trial with an immediate start AEP group and a delayed-start SP, followed by AEP group. The SP group will act as the control group for the first 6-weeks of intervention at which time the primary outcome will be evaluated for comparison of stretching versus aerobic exercise. The study design and assessment schedule is outlined in Fig. 1. The trial protocol is registered on clinicaltrials.gov (NCT03895450).

### Study group

Subjects will be eligible to participate based on the following inclusion criteria: 1) aged 18–65 years; 2) diagnosis of mTBI based on American Congress of Rehabilitation Medicine criteria [28] 3) diagnosis of PPCS based on ICD-10 postconcussional syndrome criteria [29] for a minimum of 3 months to a maximum of 5 years; 3) exercise intolerance, described as acute exacerbation of post-concussive symptoms with exercise; 4) maintenance of a stable pharmacological regiment for a minimum of one month prior to intervention start with the requirement of maintaining this regiment for the duration of the trial. Exclusion criteria includes past medical history of other neurological condition or psychiatric condition (other than depression and/or anxiety), a prior moderate or severe traumatic brain injury, or a history of cardiopulmonary or chronic musculoskeletal condition.

### Study setting and recruitment

Participants will be recruited from the Calgary Brain Injury Program at Foothills Medical Centre, University of Calgary Sports Medicine Centre concussion clinic, Calgary Pain Program and a private physiotherapy clinic in Calgary, AB, CAN where treating physicians will obtain consent to contact. Once a participant is referred and has provided consent to contact, they will be contacted by a member of the study team to discuss participation. If the individual is interested and meets inclusion criteria, an initial assessment will be scheduled. Prior to beginning the initial assessment, participants will complete a digital informed consent.

Participants may consent for the research team to use magnetic resonance imaging (MRI) data for future studies/analysis. The consent form reads as follows: “In some cases, we may wish to use your MRI data for other research studies by members of this study team. Please check one of the boxes below: a) I do not consent to having my MRI used in other research studies in addition to this study; b) I do consent to having my MRI used in other research studies”. Following checking either option a or b, a digital signature (initials) are obtained.

### Blinding and randomization

Each participant will be randomized following screening and enrollment in the study. Participants will be randomized to either the immediate start (AEP) or delayed-start group (SP + AEP) using a computer-generated randomization sequence with block sizes of 10. Allocation details are kept in opaque envelopes and revealed sequentially. Due to the nature of the intervention only the data analyst will be blinded.

### Interventions

#### Treatment arms

Once screening is complete and a participant is enrolled, they will be randomized to either begin the aerobic exercise protocol immediately (AEP, *n* = 28), or begin the aerobic exercise following a precursor placebo-like stretching protocol (SP, n = 28). Participants in both groups will complete a digital daily exercise diary for the duration of the protocol.

#### Aerobic exercise intervention

Participants are asked to complete approximately 30 mins of aerobic exercise, 5x/week for 12-weeks. The assigned exercise prescription will be 70–80% of max HR as achieved during exercise testing. Exercise prescriptions will be updated every 3-weeks. HR will be monitored by participants during exercise using a PolarH10 (Polar Canada) HR monitor, which connects to the *Polar Beat: Run & Fitness* app, allowing for ease of monitoring. Exercise location and mode of exercise (treadmill, outdoor walking or jogging, stationary bike, swimming or elliptical) will be at the discretion of participants and vary depending on exercise prescription and access. Mode and location of exercise will be discussed with a member of the study team at the initial and follow-up assessments and ability to continue will be assessed.

Exercise prescriptions will be based on HR at point of symptom exacerbation as determined using the Buffalo Concussion Treadmill Test (BCTT). The BCTT is an incremental treadmill exercise test following a modified Balke protocol [30], as first described by Leddy and colleagues, which progresses to the first sign of symptom exacerbation. All exercise testing will be under the supervision of a certified exercise physiologist. During all assessments a physician will be on call and will be paged in the unlikely event of an emergency. If there is a medical emergency requiring life supporting immediate medical attention 911 will be called. All members of the study team are first aid and CPR certified. A PolarH10 HR monitor will be worn for the duration of the test. Briefly, treadmill speed will be set at 1.7mph at 0.0% incline for a 4-min warm-up. The protocol begins with speed set at 3.3mph at 0.0% incline for the first minute. Each minute thereafter incline will increase by 1% while the speed is maintained. Rating of perceived exertion (RPE) and overall symptom burden are collected every minute. The test is terminated upon symptom exacerbation, defined as an increase of ≥3 points on a 0–10 visual analogue scale (VAS) [30] or at the point of voluntary exhaustion (9 on a 0–10 Borg-RPE scale [31, 32]). If the participant reaches the maximum incline and is able to continue, speed will be increased by 0.4mph each subsequent minute. Testing will be postponed if the participant has a pre-test overall symptom burden ≥7 on the VAS.

#### Stretching intervention

Participants are asked to complete a 30 min, low-intensity stretching program 5x/week for 6 weeks. A detailed program with each day’s stretches (written and figure format) will be provided. None of the stretches have neck involvement as not to exacerbate neck pain or headache symptoms. All stretches will be demonstrated by a member of the study team at the initial assessment. Participants are instructed to not exceed 50% of age predicted max HR during stretching. HR will be monitored by participants using a PolarH10 HR monitor.

### Measures

The baseline assessment will include participant-reported instruments to collect information on demographics, past medical history, acute rest advice they received following mTBI and self-reported measures of physical and sedentary activity. Outcomes collected at baseline, following 6-weeks and 12-weeks of intervention include symptom burden, assessments of specific symptoms, exercise intolerance and MRI, including spectroscopy, detailed below. A blood draw will also be completed at these time points along with five nights of sleep actigraphy. An electronic follow up (E-FU) will be completed at 3 and 9-weeks post-AEP completion and include specific symptom questionnaires in addition to questions regarding exercise behavior since intervention completion.

### Primary outcome measure

The Rivermead Post-concussion Symptom Questionnaire (RPQ) is a measure of symptom burden which evaluates the severity of 16 commonly experienced post-concussive symptoms [33] frequently used in mTBI and PPCS research. The suggested minimal clinically important difference (MCID) at the time of writing is 4.6pts based on the difference in scores between individuals < 3 months post-mTBI given prolonged rest advice at the time of injury compared to those not given prolonged rest advice (in line with current guidelines) [34].

### Secondary outcome measures

#### Quality of life

Quality of life will be assessed using the Quality of Life After Brain Injury Questionnaire (QOLIBRI). This questionnaire is designed for individuals having suffered a traumatic brain injury and has six subscales containing a total of 37 items [35, 36]. The first four subscales probe satisfaction with health-related quality of life, including cognition, independence and social aspects. The final two subscales address “how bothered” the individual is by emotions and physical problems.

#### Specific symptom severity measures

Symptom questionnaires will be used to assess headache, mood, anxiety, fatigue, dizziness and daytime sleepiness.

1. *Headache*: The impact of headache on daily function will be assessed with the Headache Impact Test (HIT-6, [37]). The questionnaire’s six items address pain severity, headache impact on activities of daily living, psychological distress and cognitive functioning. Greater total scores are indicative of greater impact with the MCID being a change of 8pts [38].
2. *Mood:* The Patient Health Questionnaire (PHQ-9) will be used to assess the presence and frequency of depressive symptoms [39]. This tool has been validated and is commonly used in individuals with mild to severe TBI [40, 41]. The MCID is 5pts [42].
3. *Anxiety:* The Generalized Anxiety Disorder Scale (GAD-7) is a commonly used measure of anxiety in the mTBI population [43].
4. *Fatigue*: The Fatigue Severity Scale (FSS) is a commonly used brief assessment of fatigue with greater scores indicating more severe fatigue [44].
5. *Dizziness*: The Dizziness Handicap Inventory (DHI) is a 25 item assessment of the handicapping effects imposed by vestibular system disease [45]. Items are grouped into three subscales representing the functional, emotional and physical aspects of dizziness and unsteadiness.
6. *Daytime sleepiness*: The Epworth Sleepiness Scale (ESS) rates the chance of dozing off in 8 different situations [46]. This measure has been widely used in TBI cohorts to assess sleep changes after head injury [47–50].

#### Sleep parameters

Wrist worn actigraphy (MotionWatch 8© [MW8]; CamNTech) will be used to monitor sleep parameters, such as duration and sleep onset latency, pre and post intervention. Sleep diaries will also be collected. It has been suggested that actigraphy assessment is a useful supplement to self-report measures of sleep following TBI [51]. The MW8 has previously been used for sleep analysis in individuals with bipolar disorder [52] and stroke [53]. In older adults, subjective sleep quality has been found to differ from objective measures, such as actigraphy, supporting a best practice of using both measures [54].

#### Exercise intolerance

Exercise intolerance will be measured using the BCTT to assess change in symptom burden (rated on 0–10 VAS) with an increase in HR and exercise intensity. Ability to complete a greater number of stages during the BCTT indicates decreased exercise intolerance and ability to return to play/activity.

#### Blood biomarkers

Blood biomarkers of exercise and recovery will be evaluated to probe underlying mechanisms of response to AEP and SP. Blood samples (7 mL total) will be collected under clean technique from inner elbow at baseline, 6-weeks and 12-weeks of intervention. Markers of overall brain health and response to exercise, specifically brain-derived neurotropic factor, will be analyzed according to validated methods provided by Meso Scale Discovery (SD; Gaithersburd, MD, USA) on their electrochemiluminescence platform. Cytokines will be evaluated to further understand whether there is a chronic inflammatory response to injury in this cohort and whether that is affected by the intervention. Cytokine concentrations will be determined using the Bio-Plex Pro™ Cytokine, Chemokine, and Growth Factor Assays from Bio-Rad (Herkules, CA, USA). Samples will be assayed as instructed by the manufacturer. The plates will be read on a Luminex 200 apparatus (Applied Cytometry Systems, UK). Samples will be acquired and analysed using BioPlex Manager 6.0. Should there be a coefficient of variance of 20% or higher between two replicates, data points will be considered missing. Telomere lengths (TL) will be evaluated as they are known to be neuroprotective and resiliency promoting. In a rodent model, TL has been shown to decrease following mTBI [55]. DNA will be isolated from the red blood cell clot for telomere analysis. TL measurement using qRT-PCR will follow the protocol established by Cawthon [56].

#### Brain imaging outcomes

Magnetic resonance brain imaging will be performed to investigate changes with the AEP and SP to better understand recovery or persistence of symptoms. The imaging protocol includes a T1-weighted structural acquisition, 1H magnetic resonance single voxel magnetic resonance spectroscopy (MRS), arterial spin labeling (ASL) to quantify tissue perfusion, and resting-state functional MRI. The MRS voxels will be in the sensorimotor cortex and the anterior cingulate. Of specific interest are the MRS biomarkers γ-aminobutyric acid (GABA) and glutathione that will be simultaneously measured using a new magnetic resonance spectroscopy method (HERMES, Hadamard Encoding and Reconstruction of MEGA-Edited Spectroscopy) [57]. Glutathione, an antioxidant that combats oxidative stress, is hypothesized to be reduced following brain injury and is expected to change with aerobic exercise. GABA is an inhibitory metabolite which is modulated with brain plasticity. It has been proposed that increased GABA impedes recovery from mTBI [58]. Additional metabolites of interest from MRS (that will be measured using a point-resolved spectroscopy, PRESS) are glutamate, a marker of excitability, and N-acetyl aspartate (NAA), a marker of neuronal health.

### Attrition and adherence

Participants will be withdrawn from the study if there is a change in medications or if they are unable to attend follow ups. Adherence to aerobic exercise and stretching protocols will be tracked using online daily diaries (using Research Electronic Data Capture [REDCap]) sent out via email. REDCap is a secure, web-based application for data capture. Diaries will be completed by all participants for the duration of the study and submitted on their mobile device. Daily diaries will track symptomatology before and after prescribed exercise, physical activity beyond what is prescribed, daily symptom burden rating on a VAS and medication usage. In the event that the activity diary is not being completed, reminders will be sent out by the study team. A record will be kept of sent reminders.

### Data management

Participants will be assigned a study ID number at the time of randomization by which they will be identified within the research team. Study data will be entered into a highly secure electronic REDCap database which requires double password protection to enter. Only research team members will have access to this database. Participant information or other data transmitted through USB will be encrypted. USB transfer of information will only be used when absolutely necessary and will be password protected. Paper copies of any data or participant information will be kept in a locked cabinet in a locked room in a locked university building.

All identifying information will be removed once all study participant numbers have been assigned and all data is collected. When data is downloaded for analysis, manuscript writing or abstract preparation all identifying information will be excluded. This information will be downloaded to a password protected SPSS file for analysis. No identifiable information will be retained once data collection is complete. All data will be retained for five years following project completion in accordance with Health Canada retention guidelines.

### Sample size

A power calculation using significant outcomes for RPQ in a neurorehabilitation/exercise intervention suggested 46 participants should be included to find significant improvement, with an alpha of 0.05 and 80% power [59]. With an anticipated dropout rate of 1–5 participants per group we will recruit 56 participants for this trial.

### Analysis

The primary clinical outcome is the RPQ at 6-weeks of intervention. For the clinical outcome measures, two separate analyses will be run:

1. Outcome measures will be analyzed using two-way mixed ANOVA at baseline and 6-week timepoints. Two-way mixed ANOVA allows for analysis of group by time interaction effect. If a statistically significant group by time interaction effect is detected, indicating that group effect varies with time, or time effect varies with the group, simple effect testing will be used to analyze group effect broken down by time or time effect broken down by group. Should there be missing values, generalized estimating equation analysis will be employed.
2. Outcome measures between groups (delayed-start vs. immediate start) following the aerobic exercise intervention only will be analyzed using two-way mixed ANOVA at the three primary timepoints: baseline, 6-weeks and 12-weeks.

The HERMES MRS data will be analysed to quantify GABA and glutathione levels using Gannet3, a spectral analysis tool. The PRESS MRS data will be analyzed to quantify NAA, glutamate, creatine and choline. Metabolite levels will be compared using an ANOVA model comparing groups at each of the three timepoints (as above). Additional exploratory analyses will investigate the correlations between metabolite levels and symptom burden. Whole brain ASL data will be processed to provide quantitative maps of tissue perfusion (cerebral blood flow) and group data will be compared on a whole brain basis. If no differences are detected, regional analyses may be applied. Resting state fMRI data will investigate differences in functional connectivity between groups and time points using both independent component analysis and seed-based approaches.

### Study status

At the time of submission we are recruiting and enrolling participants in the study.

### Access to data

The principal investigator, research assistants, students and statistician colleagues who are directly involved in the study will have access to the final data set.

### Dissemination policy

Trial results will be disseminated through presentations at conferences, invited presentations and published manuscripts by study authors and contributors. The study is registered on clinicaltrials.gov. There will be no use of professional writers.

## Discussion

This study will expand our understanding of the utility of sub-symptom threshold aerobic exercise as an intervention for the treatment of PPCS as it has not previously been investigated in an adult population. The use of MRS for analysis of brain metabolites in addition to blood biomarker investigations will provide greater understanding of the pathophysiology of this patient cohort and their response to intervention. This trial will expand on previous work in the domain of adolescent SRC to evaluate this intervention in an adult PPCS population with diverse etiology of injury and chronic symptomatology. Should this trial be successful, it will be largely generalizable to the adult PPCS population. The proposed intervention is easy to implement, low cost and a novel non-pharmacological treatment option for a patient population with high rates of polypharmacy. An aerobic exercise intervention such as the one described has the potential to not only improve exercise intolerance, but also treat multiple chronic symptoms experienced by adults whose quality of life and activity has been greatly limited by PPCS.

## Data Availability

Not applicable.

## Abbreviations

AEP: Aerobic exercise protocol
ASL: Arterial spin labeling
BCTT: Buffalo concussion treadmill test
DHI: Dizziness Handicap Inventory
E-FU: Electronic follow-up
ESS: Epworth Sleepiness Scale
FSS: Fatigue Severity Scale
GABA: γ-aminobutyric acid
GAD-7: Generalized Anxiety Disorder Scale
HERMES: Hadamard Encoding and Reconstruction of MEGA-Edited Spectroscopy
HIT-6: Headache Impact Test
HR: Heart rate
MCID: Minimal clinically important difference
MRI: Magnetic resonance imaging
MRS: Magnetic Resonance Spectroscopy
mTBI: mild traumatic brain injury
MW8: MotionWatch 8©
NAA: N-acetyl aspartate
PHQ-9: Patient Health Questionnaire
PPCS: Persistent Post-concussive symptoms
PRESS: Point-resolved spectroscopy
QOLIBRI: Quality of Life After Brain Injury Questionnaire
REDCap: Research Electronic Data Capture
RPE: Rating of perceived exertion
RPQ: Rivermead Post-concussion Symptom Questionnaire
SP: Stretching protocol
SRC: Sport-related concussion
TL: Telomere length
VAS: Visual analogue scale
